# Individual Variation in Migration and Wintering Patterns of Long‐Tailed Ducks *Clangula hyemalis* From a Population in Decline

**DOI:** 10.1002/ece3.71187

**Published:** 2025-04-01

**Authors:** Thiemo Karwinkel, Ingrid L. Pollet, Sandra Vardeh, Julia Loshchagina, Petr Glazov, Alexander Kondratyev, Aleksandr Sokolov, Vasiliy Sokolov, Julius Morkūnas, Daniela J. Tritscher, Juan F. Masello, Götz Eichhorn, Helmut Kruckenberg, Petra Quillfeldt, Jochen Bellebaum

**Affiliations:** ^1^ Department of Animal Ecology & Systematics Justus Liebig University Giessen Gießen Germany; ^2^ Carl von Ossietzky Universität Oldenburg, School of Mathematics and Science Institute of Biology and Environmental Sciences Oldenburg Germany; ^3^ Institute of Avian Research “Vogelwarte Helgoland” Wilhelmshaven Germany; ^4^ Acadia University Wolfville Nova Scotia Canada; ^5^ Institute of Geography RAS Moscow Russia; ^6^ Institute of Biological Problems of the North FEB RAS Magadan Russia; ^7^ Arctic Research Station of the Institute of Plant and Animal Ecology of the Ural Branch of the Russian Academy of Sciences Labytnangi Yamal‐Nenets Russia; ^8^ Institute of Plant and Animal Ecology of the Ural Branch of the Russian Academy of Sciences Ekaterinburg Russia; ^9^ Marine Research Institute Klaipėda University Klaipėda Lithuania; ^10^ TUM School of Life Sciences, Technical University of Munich Freising Germany; ^11^ Department of Evolutionary Population Genetics Bielefeld University Bielefeld Germany; ^12^ Department of Biological Sciences University of Venda Venda Republic of South Africa; ^13^ Vogeltrekstation—Dutch Centre for Avian Migration and Demography Netherlands Institute of Ecology (NIOO‐KNAW) Wageningen the Netherlands; ^14^ Michael‐Otto‐Institut Im NABU Bergenhusen Germany; ^15^ Institute for Waterbird and Wetlands Research (IWWR) e.V. Germany Verden Germany

**Keywords:** Baltic Sea, bird migration, conservation, geolocator, long‐tailed duck, repeatability

## Abstract

The population of long‐tailed ducks *Clangula hyemalis* has declined dramatically since the 1990s at the species' most important wintering area, the Baltic Sea. It is unclear if this represents a real population decline at the flyway level or merely a northward shift in the wintering range, with part of the population moving from the Baltic Sea to rarelysurveyed ice‐free Arctic waters. To investigate wintering area choice and individual repeatability, we deployed light‐level loggers on female long‐tailed ducks at three breeding sites in the Western Russian Arctic across two annual cycles, from 2017 to 2019. We obtained data from 94 year‐round migration tracks (78, 14 and 2 from each breeding site) from 65 females. Females moved from freshwater breeding sites to mostly marine post‐breeding sites after wing moult. For wintering, the majority of the birds (94%) migrated to the Baltic Sea, while the rest overwintered in the White and Barents Seas. Spring migration involved staging at marine sites in the Arctic Ocean for most birds. Individual repeatability scores were high for longitudes of wintering sites, departure dates from breeding and wintering sites, and low for arrival dates at breeding and wintering sites. Therefore, our results suggest that the observed decline in the long‐tailed duck wintering population in the Baltic Sea is unlikely the result of a shift in wintering range within individuals, so that a real decline in the population size remains the most parsimonious explanation. High repeatability values indicate that the substantial variation in wintering sites throughout the Baltic Sea is clearly attributable to between‐individual variation rather than within‐individual variation across years. Still, addressing the underlying causes of population decline remains a challenge for this Arctic‐breeding species.

## Graphical abstract

The population of the long‐tailed duck ( *Clangula hyemalis* ) in its primary wintering area, the Baltic Sea, has significantly declined since the 1990s, potentially due to a northward shift in wintering range to ice‐free Arctic waters. We tracked female ducks with light‐level loggers from their breeding sites, discovering that most birds (94%) still overwinter in the Baltic Sea, with high individual repeatability. This consistency in behaviour suggests the population decline is likely due to a real reduction in numbers rather than individual range shifts, indicating the need for further investigation into the causes of this decline.

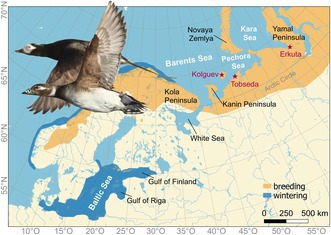

## 1 Introduction

Migratory species use a wide range of habitats and face various environmental conditions and threats throughout their annual cycle (Mallory 2006; González‐Solís et al. 2007; Schmaljohann et al. 2012; Fox et al. 2015). To design conservation measures appropriately, detailed knowledge of the movements of migratory species is crucial (e.g., Runge et al. 2014; Bauer et al. 2016). Yet, migratory behavior is not static and animals can adjust their movements and timing to adapt to changes in their environment (Anderson et al. 2013; Rickbeil et al. 2019). Climate change, alongside human activities, increasingly affects migratory animals at different stages of their annual cycle (Fox et al. 2015). To make conservation measures effective in the long term, especially for long‐lived animals, conservation efforts also need to consider plasticity, as it predicts the possible rate of behavioral change (Snell‐Rood 2013; Stamps 2016).

Since 1980, various migrant diving ducks and fish‐eating waterbirds wintering in Central Europe and around the Baltic have shifted their wintering range to the North and East across Europe as winter temperatures increased (Lehikoinen et al. 2013; Pavón‐Jordán et al. 2019). With the retreat of winter sea ice in the Arctic Ocean (Cornish et al. 2022), more potential wintering habitats become available closer to the breeding grounds of Arctic breeding sea ducks. These birds are therefore likely to show northward shifts in their winter distribution, and such shifts could in turn potentially explain recent declines in traditional wintering areas (Fox et al. 2019).

One of those waterbirds, the long‐tailed duck *Clangula hyemalis* , is a sea duck with a circumpolar breeding distribution, nesting at lakes and ponds in the Arctic tundra (Robertson and Savard 2002). It generally winters in coastal waters and offshore banks of North America, Northeast Asia and Northern Europe, where it chiefly feeds on benthic crustaceans, mussels, clams and fish (Robertson and Savard 2002; Žydelis and Ruškytė 2005; Skabeikis et al. 2019). Like other ducks, long‐tailed ducks only maintain seasonal pair bonds. Females incubate and raise ducklings without males, which usually leave the breeding site before hatching (Quillfeldt et al. 2021).

The largest of four identifiable flyway populations of long‐tailed ducks breeds in Western Russia and Northern Europe. The Western Russia population alone comprises about 90% of the Europes' breeding pairs (Keller et al. 2020). The current estimate in these regions is 1.6 million individuals (Hearn et al. 2015; Wetlands International 2018). This population winters mainly in shallow offshore waters of the Baltic Sea, where overall wintering numbers have declined by 65% since the 1990s (Skov et al. 2011) and locally by up to 82% (Bellebaum et al. 2014). This decline might arise from a combination of factors operating during different phases of the annual cycle. In the Baltic Sea, direct threats include mortality from bycatch in gillnet fisheries (Žydelis et al. 2009) and from chronic oil pollution (Larsson and Tydén 2005). Rising water temperatures adversely affect major food sources during winter and spring migration, such as Baltic herring ( *Clupea harengus* ) spawn, which is consumed in spring (Žydelis and Ruškytė 2005; Polte et al. 2021) and blue mussels ( *Mytilus edulis* ; Waldeck and Larsson 2013). Blue mussels are also negatively affected by the spread of invasive fish feeding on the molluscs (Skabeikis et al. 2019). With the decrease of preferred prey items, long‐tailed ducks may resort to consuming prey with a lower energetic value. This food shortage, in turn, may impact their survival and the body condition in which individuals arrive at their breeding locations, ultimately affecting their reproductive prospects (Bauer et al. 2008). Finally, changes in predation pressure and environmental conditions at the breeding grounds drive declines of sea ducks in Canada and Scandinavia (Iles et al. 2013; Fox et al. 2015) and possibly also in the Russian Arctic. Hence, information about areas used, especially by the egg‐producing females, during the entire annual cycle is required to inform future conservation actions.

Expanding on previous studies with a limited number of birds from just one breeding site and just  one year (Karwinkel et al. 2020; Quillfeldt et al. 2021), here we analysed the spatiotemporal patterns of migrations and wintering locations of female long‐tailed ducks from three different breeding sites in the Western Russian Arctic over multiple years. Females were selected because they are faithful to their breeding grounds, making it possible to recapture them and recover the data‐loggers. Like most sea ducks, long‐tailed ducks tend to form pairs on the wintering grounds, with males following their partners to the breeding sites, which makes them less likely to return to the same breeding area (Bartzen et al. 2017). We use this multi‐year and multi‐site study to find out whether the decline of winter numbers in the Baltic Sea is due to a true decline of the breeding population or rather a shift in the wintering range out of the Baltic Sea. Using repeatability measures allows us to investigate whether the variation in migratory traits, as for example observed for wintering longitude (Karwinkel et al. 2020), originates from within‐individual (between years) or between‐individual differences.

## 2 Methods

### 2.1 Study Sites

**FIGURE 1:**
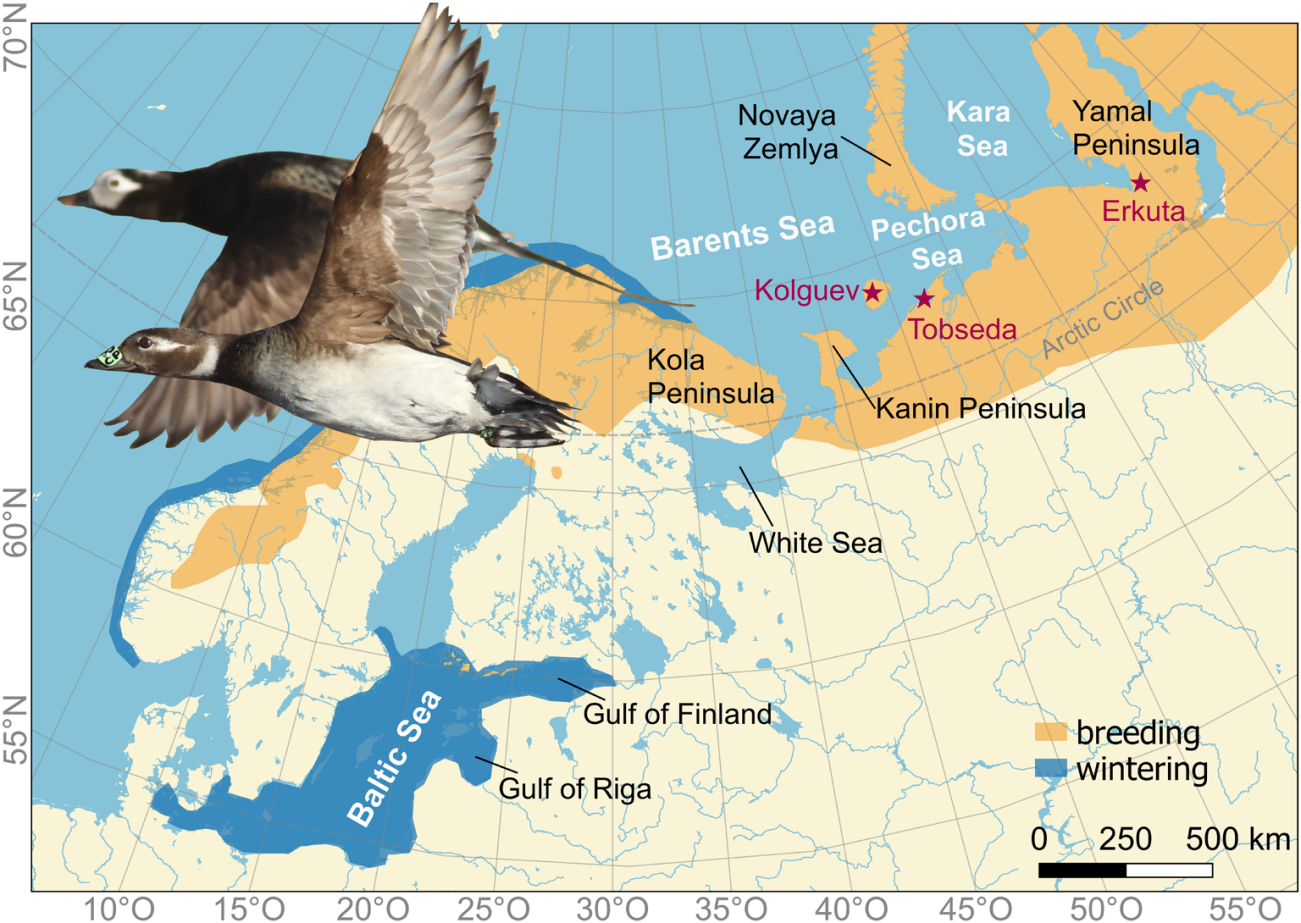
Distribution of long‐tailed ducks (orange: breeding; dark blue: wintering; BirdLife International and Handbook of the Birds of the World 2024). Locations of field sites (red stars) and major areas mentioned in the text. The photo inset shows a long‐tailed duck couple in flight from Tobseda, with the female in front carrying a nasal saddle and a geolocator attached to the metal band around the tarsus. Photo: TK.

This study took place at three field sites in the Western Russian Arctic (Figure 1, Table 1), where 90% of the European population of long‐tailed ducks breed (Keller et al. 2020). Kolguev Island (69°07′ N 48°45′ E) and Tobseda (68°35′ N 52°19′ E) are located at the Barents Sea in the Nenets Autonomous District, presumably the most important breeding region of the species in Europe (Keller et al. 2020), representing an island location and a coastal mainland tundra habitat, respectively. On the 5020 km^2^ large island of Kolguev, 72 km off the mainland, long‐tailed ducks breed inland at many small tundra lakes (Glazov et al. 2021). Tobseda is located on a peninsula at the entrance to Kolokolkova Bay, where ducks breed mainly on small ponds in coastal sand dunes, and adjacent tundra lakes of various sizes. Erkuta (68°14′ N 69°13′ E) is located close to the confluence of Erkuta and Payuta rivers in the south‐western Yamal Peninsula attached to the Eurasian Continent, about 25 km from Baydaratskaya Bay, where ducks breed on tundra lakes. With the choice of these field sites, we are confident that we gain a representative sample of the flyway population.

**TABLE 1:** Summary of light‐level geolocators (GLS) deployed on female long‐tailed ducks at three field sites in the Russian Arctic and numbers of individuals recaptured the following years.

Site	GLS deployed in 2017	GLS recovered in 2018	GLS deployed in 2018	GLS recovered in 2019	Number of birds tracked	Number of birds tracked for 2 years
Erkuta	8	0	25	1	1	1
Kolguev	48	19	52	46^a^	50	27
Tobseda	0	0	27	14	14	0
Total	56	19	104	60	65	28

^a^ Including a dead bird found on the German Baltic Sea Coast in May 2020 and another bird recaptured in July 2021.

### 2.2 Geolocator Deployment and Retrieval

Female long‐tailed ducks were caught on breeding lakes (and also on a river in Erkuta) using either mist nets or floating, slightly weighted monofilament gillnets (Ferguson 1990) with mesh sizes of 45 mm. Once captured, ducks were marked with metal rings and either with individually marked 38 × 17 mm nasal plastic tags (Rodrigues et al. 2006; Figure 1) or with unmarked nasal plastic discs of 14 mm diameter (Lyakhov 2016) attached with 1.2 mm fishing line to allow re‐sightings and targeted re‐captures.

Two types of light‐level geolocators, that is, global location sensors (GLS), were deployed during this study. During the 2017 breeding season, we deployed Intigeo C330 (17 × 19 × 8 mm, 3.3 g, Migrate Technology, Cambridge, UK) with a battery life of 5 years on 56 female long‐tailed ducks between 10 June and 11 August (Table 1). In 2018, we deployed 32 C330 geolocators and 72 C65 models (14 × 8 × 6 mm, 1.0 g, Migrate Technology, Cambridge, UK). These latter geolocators were smaller and were designed to have a battery life of at least 2 years. Geolocators were attached to the metal ring using a small UV‐resistant cable tie. Geolocators and attachment weighed 3.5 g (C330) and 1.2 g (C65), which represent 0.5% and 0.2% of the average body weight, respectively (weight of females tagged: 686.6 g ± 82.3 g, *n* = 119).

Ambient light level was sampled every minute with the maximum value stored for every 5‐min period. Additionally, geolocators were programmed to sample immersion in water and conductivity every 30 s. Relative immersion values ranged from 0 (continuously dry) to 480 (continuously under water), stored every 4 h, and relative conductivity values ranged between 0 (dry) and 128 (maximum conductivity), maxium value stored every 4 h. Calibration tests in the lab with several saltwater solutions at different temperatures showed that conductivity values > 115 corresponded to sea water, while values between 100 and 115 corresponded to brackish water as in the Baltic Sea (Karwinkel et al. 2020). Temperature was sampled every 5 min, and minimum, maximum, and mean temperatures were stored every 4 h.

During the 2018 breeding season, we re‐captured tagged individuals, recovered the geolocators, and deployed new ones on 18 of these birds, and captured additional birds. In the 2019 breeding season, we re‐captured tagged individuals and removed nasal tags along with geolocator devices. Two more geolocators from Kolguev were retrieved until 2021 (Table 1).

### 2.3 Data Processing

All raw light data (‘.lux’‐files) and environment data (conductivity, temperature, wet counts; ‘.deg’‐files) together with the corresponding bird ringing sheet are available in the supplemental for future re‐analysis. Whenever the geolocator was still running during recapture, the readout programme compared the timestamp of the geolocator with the computer and adjusted the timestamp of the data according to the geolocator's time drift (datafiles marked as ‘driftadjusted’). The raw light data were then processed through the IntiProc software v. 1.03 (Migrate Technology), which uses the ‘*coord*’ and ‘*getElevation*’ functions from the ‘GeoLight’ package v. 2.0 (Lisovski and Hahn 2012) in R version 2.12.1 (R Core team 2019). We used a light threshold of 2 and a sun angle of −5.2 (angle corresponding to sunrise and sunset based on pre‐deployment calibration in central Germany, as the sun was not going below the horizon by the time we reached the field sites). We visually inspected all the twilight events and manually removed erroneous twilight events (rapid successions of sunrise and sunset events within a 24 h period), mainly caused by shading of the geolocator. Because the three field sites are located at latitudes with constant daylight during part of the breeding season (end of May through end of July), positions could not be calculated from light data during this period. Locations were plotted on a map and filtered manually by removing subjectively erroneous and non‐plausible locations. Locations obtained with this method are further referred to as ‘GeoLight method’.

The twilight events obtained from the IntiProc software (i.e., from the integrated GeoLight‐package) were additionally processed using the ‘probGLS’ package (Merkel et al. 2016) in the R environment v.3.6.1. This package, designed with seabirds in mind, integrates geolocator‐measured sea‐surface temperature (SST) with remotely‐sensed satellite sea‐surface temperature (SST) and sea‐ice cover, downloaded from the National Oceanic and Atmospheric Administration (NOAA) website (https://www.esrl.noaa.gov/psd/data/gridded/data.noaa.oisst.v2.highres.html) to estimate the locations of the birds using an iterative forward step selection process. It also considers conductivity data, a land mask and the known travel speed of the studied species (Karwinkel et al. 2020). Locations obtained with this method are further referred to as ‘probGLS method’.

### 2.4 Definition of Breeding Status

Breeding status of tagged females was assessed using light and temperature recordings. This was feasible for those yearly cycles where the breeding time was recorded before the post‐breeding stage (*n* = 37), but most birds were caught after breeding time during wing moult. Summer above the Arctic Circle is characterised by continuous daylight; thus, longer periods of darkness indicate that the bird has been sitting on land, shielding the geolocator from light with the body feathers (Tertitski et al. 2021). Dark periods over large parts of the day together with logger temperatures high above ambient temperature suggest the female is incubating. We assumed incubation when light was below 4 lx and temperature was above 20°C at the same time. With these criteria, we distinguished non‐breeders not showing any signs of incubation (‘N’ in data table ‘migration.characteristics.LTD.xlsx’), successful breeders showing constant incubation over a minimum of 23 days and then ending sharply (‘S’) and unsuccessful, that is, failed, breeders showing signs of incubation over less than 23 days (‘F’).

### 2.5 Definition of Movements

We identified the departure from the ‘breeding stage’ as the time when we detected a shift in conductivity from freshwater to seawater, occasionally occurring together with a change in longitude. We defined the following ‘post‐breeding stage’ as the period, when ducks left their breeding sites for coastal or marine areas. The post‐breeding stage included the autumn equinox, a period of several weeks when geolocators cannot provide reliable latitude, and we had to rely on longitude estimates only. ‘Autumn migration’ was detected by a rapid change in longitudinal values and ended when longitudinal changes were less than about 10° and conductivity values remained constant over several days. The end of autumn migration started the ‘wintering stage’, where longitude and conductivity values remained constant until the onset of spring migration. We defined the start of spring migration as a rapid change in longitudinal and conductivity values, which means leaving the Baltic Sea for most birds (see Figure S1 for an example). Spring migration ended when conductivity and longitudinal values showed constancy again. The period following the spring migration was defined as ‘pre‐breeding stage’, and lasted until the bird stayed constantly in freshwater, where the breeding stage started again. For all stages, we manually assigned the start and end times and calculated their mean longitude and latitude (from GeoLight method). These data are available in the data set ‘migration.characteristics.LTD.xlsx’.

### 2.6 Repeatability Analysis

We calculated the intra‐individual repeatability factor *R* using the function ‘*rpt’* in the package ‘rptR’ (Stoffel et al. 2017). The repeatability represents the proportion of the total phenotypic variance of a trait (e.g., migratory departure dates) in the population that can be attributed to variation among individuals (see Stoffel et al. 2017 for more details, including formulas). *R* can vary in the range 0–1. Ecologically speaking, high repeatability values indicate a more consistent behaviour within individuals relative to higher between‐individuals variation (Franklin et al. 2022). The repeatability factor *R* is the proportion of total variation in a trait that can be explained by between‐individual variation and therefore represents the constancy of phenotypes within individuals (Nakagawa and Schielzeth 2010).

Repeatability was calculated for the following traits, similar to Kürten et al. (2022): departure date from breeding area, duration of post‐breeding stage, longitude (from GeoLight analysis) of post‐breeding stage, arrival date at wintering stage, mean longitude in every winter month from November until April (from GeoLight analysis), departure date from wintering stage, duration of pre‐breeding stage and arrival date at the breeding area. Longitude values were chosen because positions inferred from geolocators are more accurate in longitude than in latitude. Longitude of the pre‐breeding stage was not included as it contains insufficient data due to the Arctic constant daylight. Dates were transformed into Julian day (1 January = 1, continued). For the first three traits, we included the breeding status of the previous breeding stage as a cofactor, where year was used as a cofactor for the rest of the repeatability models.

## 3 Results

Of the 142 females tagged during the 2017 and 2018 breeding seasons, 65 (46%) were recaptured (Table 1). In total, 27 birds were tracked for two years and one for 3 years. Thus, 94 different year‐round movements (seven incomplete due to tag failure) were collected from 65 individuals for a total of 31,238 days. The weight of females at the time of deployment did not influence the individual's recapture (*F* = 0.34, *p* = 0.55). Of the 80 geolocators recovered, 73 worked for the full duration of the deployment. A total of four geolocators ended recording during the first winter (between 25 February and 31 March), three more geolocators stopped working on 6 May, 4 July 2019 and 30 September 2020, respectively. We obtained 55,878 locations for the GeoLight Method (see dataset ‘LTD_GeoLight.xlsx’) and 53,006 locations for the probGLS method (see dataset ‘LTD_probGLS.xlsx’).

### 3.1 Departure From Breeding Stage and Post‐Breeding Stage

**FIGURE 2:**
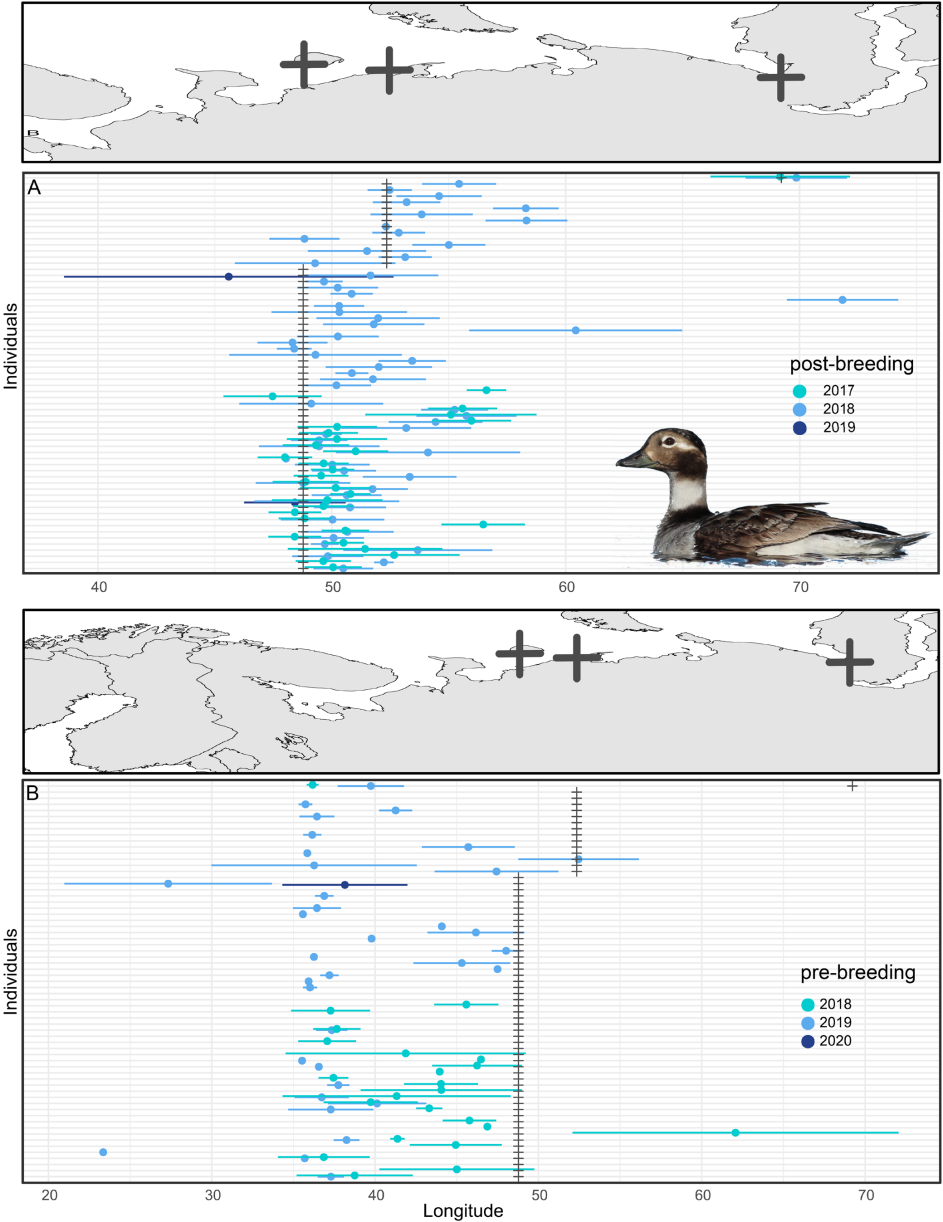
Longitudes (mean ± SD) of post‐breeding (A) and pre‐breeding (B) staging areas of migrating female long‐tailed ducks from the probGLS model. A condensed map is added above each graph for reference of longitudes (see Figure 1 for detailed map). Plus symbols (+) indicate breeding areas on maps or in the longitude plot. A version including ring numbers can be found in the Appendix (Figures S4 and S5). Inset shows a female long‐tailed duck in breeding plumage. Photo: TK.

In both 2017 and 2018, females left their breeding sites between 25 July and 11 October with no difference in median departure dates between the years (Wilcox‐test: *W* = 1006.5, *p* = 0.524, *N* = 92; Table 2). The majority of tagged individuals stayed near their breeding sites or moved eastwards inside the Pechora Sea or to the Novaya Zemlya Archipelago (Figure 2A), while only one female from Kolguev flew as far as 71° E, indicating a place off the Yamal Peninsula. Birds that successfully hatched a clutch departed significantly later (24 September ±2 d, *N* = 4) than birds that incubated unsuccessfully (30 August ±21 days, *N* = 12; Wilcox‐test: *W* = 44, *p* = 0.018) or did not breed at all (7 September ±13 days, *N* = 21; Wilcox‐test: *W* = 79, *p* = 0.008).

**TABLE 2:** Phenology of life‐history stages (mean ± standard deviation) of female long‐tailed ducks from the Russian Arctic between 2017 and 2019.

Trait	Year cycle 2017–2018	Year cycle 2018–2019
Breeding site departure	2017‐09‐10 ± 12 days	2018‐09‐08 ± 15 days
Post‐breeding duration	31 ± 13 days	28 ± 15 days
Wintering stage arrival	2017‐10‐18 ± 9 days	2018‐10‐17 ± 13 days
Wintering duration	210 ± 8 days	211 ± 11 days
Wintering stage departure	2018‐05‐16 ± 5 days	2019‐05‐16 ± 5 days
Pre‐breeding stage duration	22 ± 7 days	16 ± 6 days
Breeding site arrival	2018‐06‐07 ± 9 days	2019‐06‐03 ± 6 days
*N*	31	61

*Note:* A yearly cycle starts and ends with the breeding stage.

After the breeding stage, the birds stayed at their post‐breeding staging sites for about a month (Table 2). Afterwards, all tracked birds moved in a southwesterly direction to reach their wintering areas. For 43 tracks of 36 birds wintering in the Baltic, autumn migration included stopovers of 2 days–28 days (average 8 days ± 7 days) from the White Sea to the Kanin Peninsula (30°–45° E). One bird stopped over off the coast of Norway (according to the locations from the probGLS method) for 19 days until 16 November 2018 (ES030047).

### 3.2 Winter

**FIGURE 3:**
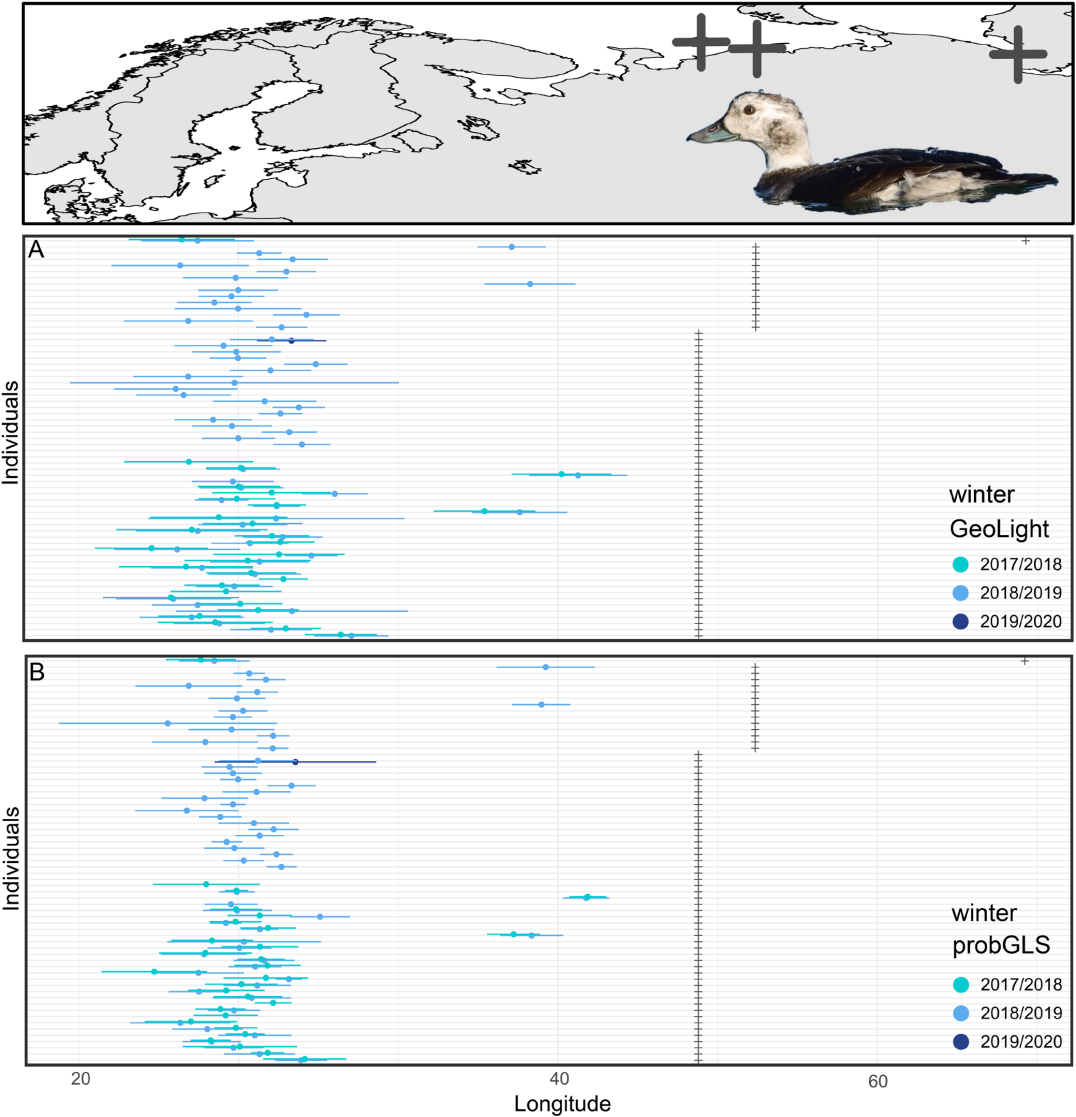
Longitudes (mean ± SD) of wintering stage from the (A) GeoLight and (B) the probGLS model of female long‐tailed ducks. A condensed map is added above for reference of longitudes (see Figure 1 for detailed map). Plus symbols (+) indicate breeding longitude of each individual. A version including ring numbers can be found in the appendix (Figures S6 and S7). Inset shows a female long‐tailed duck in wintering plumage. Photo: TK.

Female long‐tailed ducks arrived at their wintering grounds between 17 September and 19 November and remained there for 211 ± 10 days (range 179–232 days; Table 2). Two birds from Kolguev and two birds from Tobseda wintered in the White Sea or the Barents Sea, resulting in a total of six winter seasons of four individuals or 6% of the entire sample (Figure 3). Those Arctic Sea wintering sites were also confirmed by the water conductivity values (Figure S2). All remaining birds (94%) wintered in the Baltic Sea. By using the package ‘probGLS’, a land mask was applied, forcing the locations on water. During the winter months, we observed higher latitudes with this method than with the ‘GeoLight’ method during several months, so mapping of locations within the Baltic Sea would not be meaningful. We obtained 2 years of migration data for 28 birds (27 from Kolguev, 1 from Erkuta, Table 1). All birds wintered either inside (26 birds) or outside (2 birds) of the Baltic Sea in both years (Figure 3, Figure S2).

### 3.3 Pre‐Breeding Stage and Return to the Breeding Area

Female ducks initiated their spring migration around mid‐May in both years (Table 2). Spring migration included the pre‐breeding stage periods of up to 32 days, with only two birds apparently moving non‐stop to their breeding areas (ES030046, ES033639). Birds moving out of the Baltic Sea headed for marine staging sites presumably in the White and Barents Seas (Figure 2B) before moving into their breeding grounds. Birds tracked over both seasons arrived on average 4.5 days earlier at their breeding sites in 2019 than in 2018, with only six of 26 birds arriving on the same day or later.

### 3.4 Repeatability of Migratory Characteristics

**FIGURE 4:**
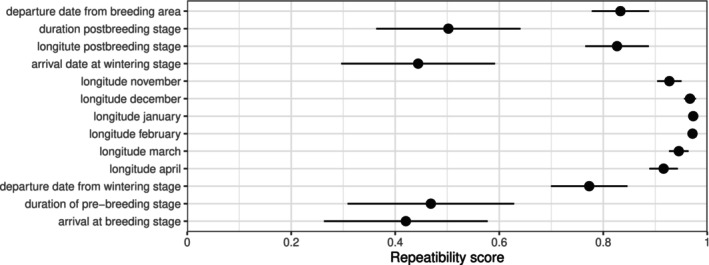
Intra‐individual repeatability of migratory traits in tracked female long‐tailed ducks. Dots show repeatability scores with standard error from the repeatability model. See Table S1 for model results and Figure S3 for a more detailed version including sample size.

The variation in wintering sites, represented by the monthly average longitude, can be mainly attributed to between‐individual variation and not to within‐individual variation, indicated by a high repeatability score (*R* > 0.9; Figure 4, Table S1, see methods for definition of repeatability). The departure date from the wintering site was also highly repeatable (*R* > 0.75), whereas arrival dates at the wintering sites showed a much lower repeatability (*R* < 0.5). The departure and arrival dates at the breeding sites show a similar pattern: whereas departure from the breeding sites is highly repeatable with *R* > 0.8, the arrival at the breeding sites only shows moderate to low repeatability of *R* < 0.5. When including the breeding status (successful breeding, failed breeding, non‐breeding) in the repeatability model, the repeatability value for the departure date from breeding stage is slightly higher (Figure S3), suggesting the breeding status to have an influence on the departure date (see also section 3.1 ‘Departure from breeding stage and post‐breeding stage’). The repeatability of the longitude of the breeding stage was not calculated, as birds were captured and recaptured at the same site, which would bias the repeatability to *R* = 1. Repeatability of the duration of the intermediate stages, the post‐ and pre‐breeding stages, was moderate (*R* ~ 0.5), whereas the post‐breeding location, represented by longitude, was highly repeatable (*R* > 0.8).

## 4 Discussion

Using lightweight light‐level geolocators, we were able to quantify the timing of migration and location of wintering grounds of female long‐tailed ducks without risking impacts of implanted transmitters on the fitness of females or their readiness to return to the breeding grounds. Tracking female long‐tailed ducks over the full annual cycle over consecutive years from multiple breeding sites provided valuable information on the key areas for the species and the origin of variation in timing and locations. Specifically, the Baltic Sea remains the most important wintering area for this flyway population. Further, variation in migratory traits, especially wintering longitude, can be predominantly attributed to between‐individual variation and not to within‐individual variation between years.

### 4.1 Moult and Autumn Migration

At the end of the breeding season, mostly in August, long‐tailed ducks go through a pre‐basic moult, including the moult of their flight feathers (i.e. remigial moult). During that time, they are flightless for 3–4 weeks (Robertson and Savard 2002; Flint et al. 2004). Successfully breeding females undergo their flight feather moult while tending their ducklings. Other birds may use large freshwater lakes, wetlands, or coastal marine habitats (McLaren and McLaren 1982; Robertson and Savard 2002). Most of the females in our study remained close to their breeding sites until late August or early September, with only six tracks produced by four birds (6% of the tracks) showing an early departure by or before 4 August, which is before primary moult. Most of the adult females caught and measured during August were unable to fly and had growing wings shorter than 170 mm (fully grown wing length: 201–227 mm), indicating that females regularly moult their primaries in our study areas. Moulting of females near their breeding sites was confirmed by the satellite tracking of five females from Kolguev (Quillfeldt et al. 2021), whereas in Alaska 13 out of 25 females undertook a summer moult migration over > 200 km from their breeding grounds until early August (Petersen et al. 2003). Unlike females, males from Kolguev tracked with satellite transmitters left the island in mid‐July for moulting sites in the Kara Sea > 300 km east of Kolguev (Quillfeldt et al. 2021). In our study, the first departures from the breeding sites towards nearby sea or brackish water for primary moult took place on 25 and 26 July 2018 in Kolguev and Tobseda, respectively.

Females departed from the breeding sites later after successful incubation. In sea ducks, successful breeding females typically undergo remigial moult later than males or unsuccessful and non‐breeding females (Savard and Petersen 2015). Two females incubated successfully in 2017 but were non‐breeders in 2018. Their departure from their breeding site and therefore probably also wing moult was 25 and 15 days earlier in 2018 compared to 2017.

During a subsequent autumn staging period, females continued to moult their body feathers either close to breeding sites or in other known moulting areas like the Novaya Zemlya Archipelago or the Kara Strait (Loshchagina et al. 2019; Sokolov et al. 2019; Quillfeldt et al. 2021). Only one female from Kolguev travelled as far as the Yamal Peninsula, where we recorded seven out of eight males from Kolguev during moult (Quillfeldt et al. 2021), leading to the speculation that this female followed males.

### 4.2 Wintering

Before the decline in numbers, about 90% of the long‐tailed ducks breeding in the western part of Russia and Northern Europe wintered in shallow waters in the Baltic Sea south of c. 60° N (Durinck et al. 1994). More recent counts suggest that this had not changed until 2011 (Hearn et al. 2015), while the remaining birds used ice‐free areas off the coast of Norway to the southern Barents Sea (Heggøy and Shimmings 2016; Loshchagina et al. 2019; Karwinkel et al. 2020). With 94% of the tracked females using the Baltic Sea and a small remaining percentage staying in the White and Barents Seas, our study is in line with former observations and does not, so far, indicate a major northward shift in wintering grounds out of the Baltic. Despite the limitations of light‐level geolocators, we could reliably distinguish birds wintering in brackish water of the Baltic from seawater elsewhere by conductivity measured simultaneously. Similarly, eight out of nine satellite‐tagged male long‐tailed ducks from Kolguev spent the winter 2019–2020 in the Baltic (Quillfeldt et al. 2021). Additionally, the sex ratio of long‐tailed ducks in the Baltic Sea is slightly male biased and is not the subject of any significant long‐term change (Larsson 2022). Therefore, it seems very unlikely that a change in the male wintering range, invisible to this study, could partly or entirely explain the decline in wintering numbers in the Baltic.

Evidence for recent temperature‐related shifts in the winter distribution of European sea ducks exists for Steller's eider ( *Polysticta stelleri* ,) where the shift occurred eastwards along the Kola Peninsula and into the White Sea as more areas have become ice‐free during winter (Aarvak et al. 2013). The White and Barents Seas are along the traditional migration route of European long‐tailed ducks and wintering birds were already known there in the 1940s (Loshchagina et al. 2019). Until today, a large‐scale shift in the winter distribution towards areas outside the Baltic Sea is unlikely to have occurred.

Because of the limited precision of positions obtained with light‐level geolocators, assigning birds to specific wintering or staging areas within the Baltic Sea was not possible. We observed notable differences in location estimates derived from the ‘GeoLight’ and the ‘probGLS’ packages. ‘GeoLight’ uses only light levels to determine positions. Although positions obtained through the ‘probGLS’ model were based on more independent information, they tended to be unrealistically far north into the Baltic Sea compared to wintering areas known from comprehensive surveys in the Baltic Sea (Durinck et al. 1994; Skov et al. 2011) or recent satellite tracking (Quillfeldt et al. 2021).

Over 20 years, the proportions of Baltic‐wintering long‐tailed ducks in Denmark, Germany and Poland dropped from 35% to 25%, while those in Swedish coastal and offshore waters increased from 33% to 38% (Skov et al. 2011), suggesting a gradual shift of concentrations within the Baltic to the northeast like in other diving waterbirds (Pavón‐Jordán et al. 2019).

### 4.3 Spring Migration and Pre‐Breeding Stage

Similar to autumn migration, spring migration and pre‐breeding included staging in marine waters of the Arctic Ocean for most individuals before moving to freshwater breeding sites (Karwinkel et al. 2020; Figure 2B). Long‐tailed ducks arrive near their breeding areas usually in May and June depending on the time of ice melt (McLaren and McLaren 1982). Given the short Arctic breeding season, it is crucial for ducks to occupy their breeding lakes as soon as snow and ice have melted to start breeding, probably resulting in the low repeatability scores of the arrival date at the breeding stage (Figure 4) due to fluctuations in the snowmelt dates in the Arctic (Perovich and Richter‐Menge 2009). Anecdotally, on Kolguev Island, the last day when minimum temperatures dropped below 0°C was about a week earlier in 2019 (30 May) than in 2018 (5 June) and average arrival dates showed a similar difference.

### 4.4 Repeatability Over the Annual Cycle

In Karwinkel et al. (2020), we observed that long‐tailed ducks were presumably distributed all over the Baltic Sea during the wintering stage, although all birds originated from the same breeding area of only a few square kilometres. With the current study including tracking the same individuals over consecutive years, we could investigate whether this variation can be explained by between‐individual variation or within‐individual variation between years. The high repeatability values in their wintering longitude, especially from December to February, clearly attribute this to between‐individual variation, which is comparable to the high site fidelity of other wintering ducks like Barrow's goldeneye ( *Bucephala islandica* ) or the common eider ( *Somateria mollissima* ; Petersen et al. 2012; Willie et al. 2020).

Notably, these high repeatability values do not necessarily mean that individuals are absolutely consistent (see absolute values in Figures 2 and 3). High repeatability also does not mean that the birds have no plasticity to adapt to environmental change, as, for example, shown by Conklin et al. (2021), where a long‐term shift in migratory timing was observed despite high individual repeatability. Long‐term shifts of migratory traits in populations, such as wintering area, can occur either by individual plasticity over time or by generational change (Franklin et al. 2022). However, our dataset is not suitable to investigate this issue, as we do not observe a shift in wintering area and as we only tracked two years per individual, which is marginal compared to the life expectancy of up to 20 years (BTO 2025) of a long‐lived species such as the long‐tailed duck.

Despite what high repeatability might imply, wintering sites of long‐tailed ducks are located in shallow waters and are prone to freeze during cold periods, thus becoming temporarily unavailable. Major offshore wintering sites in the Baltic freeze rarely. Yet, this happens regularly in the Gulf of Finland and the Gulf of Riga, as in February 2018, but did not happen in the other two mild winters (2019 and 2020) with a much lesser extent of sea ice (FMI data, https://www.eea.europa.eu/data‐and‐maps/figures/maximum‐extent‐of‐ice‐cover‐1). According to longitudes from GeoLight, seven birds out of 57 (12%) stayed in these areas east of 24° E in February and March 2019 compared to one bird out of 29 (3%) in February 2018. However, interpreting movements within the Baltic Sea with geolocator data remains speculative.

Repeatability of longitude in the post‐breeding phase and departure times from breeding and wintering areas was also high. In contrast, durations of post‐ and pre‐breeding staging periods and arrival dates at breeding and wintering grounds were only moderately repeatable. Migration is affected by weather conditions varying between years, and long‐tailed ducks may react similarly by adjusting their stopovers and subsequent arrivals accordingly, resulting in lower individual repeatability (e.g., McLaren and McLaren 1982). This pattern of more repeatable departures than arrivals is a common pattern in bird migration, as reported by a meta‐analysis (Franklin et al. 2022), although some authors criticize the comparison of repeatability measures between traits (Conklin et al. 2013).

### 4.5 Perspective

We found that the Baltic Sea is still holding > 90% of the female long‐tailed ducks in our study. Considering that the Western Russian population, where our study sites were located, makes up 90% of the overall European breeding long‐tailed ducks, this proportion strongly suggests that the decline in the Baltic Sea most probably reflects an actual change in population size, rather than a shift of wintering sites out of the Baltic Sea towards Arctic waters. Albeit only tracking females in this study, we can assume that this interpretation also holds true for the whole population. Females spend on average 7 months in their wintering grounds. While the Baltic Sea will remain an essential wintering area for the Western Russian and Northern European breeding population in the near future, the White Sea is already an important staging area during migration. Both locations play a major role in the conservation of the species, albeit at different phases of the annual cycle.

Northward shifts over longer periods have occurred in other wintering waterbirds. The wintering latitude of long‐tailed ducks inside the Baltic Sea may already change on a scale too small to be detected with geolocators. Future monitoring in the Baltic Sea should aim for a better coverage of ice‐free areas around Finland to avoid missing an increasing part of the population. With a lack of waterbird monitoring in the Arctic Ocean (Fox et al. 2019), tracking of sea ducks from their breeding grounds is currently the only tool to obtain information about future large‐scale changes in winter distribution, where we are setting a starting point with this study.

Climate change will continue to affect all sites used by long‐tailed ducks throughout the year, including staging sites in the Arctic Ocean and the Baltic Sea. Besides water temperature and ice cover (Cornish et al. 2022; Meier et al. 2022), changes will probably include other important environmental drivers like salinity and eutrophication, which in turn would affect major benthic food resources in the Baltic Sea (Fox et al. 2015; Meier et al. 2022). Though these changes and their impact on sea ducks are difficult to predict in detail, they will force the birds somehow to adapt. Long‐tailed ducks may take advantage of inter‐ and intra‐individual variation in various aspects of behaviour, as has been observed in the case of food choice in winter (Skabeikis et al. 2019; Forni et al. 2022). Whether the species' adaptive potential is sufficient to maintain current population sizes under future environmental changes remains to be seen.

## Author Contributions

**Thiemo Karwinkel:** data curation (supporting), formal analysis (lead), investigation (supporting), methodology (lead), software (supporting), validation (supporting), visualization (lead), writing – review and editing (lead). **Ingrid L. Pollet:** data curation (lead), formal analysis (lead), investigation (supporting), methodology (lead), project administration (supporting), software (supporting), supervision (lead), validation (supporting), visualization (supporting), writing – original draft (lead), writing – review and editing (lead). **Sandra Vardeh:** data curation (lead), investigation (lead), methodology (equal), project administration (lead), supervision (lead), writing – review and editing (equal). **Julia Loshchagina:** investigation (equal), writing – review and editing (supporting). **Petr Glazov:** investigation (equal), writing – review and editing (supporting). **Alexander Kondratyev:** investigation (equal), writing – review and editing (supporting). **Aleksandr Sokolov:** investigation (equal), writing – review and editing (supporting). **Vasiliy Sokolov:** investigation (equal), writing – review and editing (supporting). **Julius Morkūnas:** investigation (equal), writing – review and editing (supporting). **Daniela J. Tritscher:** formal analysis (supporting), investigation (equal), methodology (supporting), writing – review and editing (supporting). **Juan F. Masello:** funding acquisition (supporting), investigation (equal), writing – review and editing (supporting). **Helmut Kruckenberg:** conceptualization (equal), funding acquisition (equal), investigation (equal), resources (supporting), writing – review and editing (supporting). **Götz Eichhorn:** investigation (equal), resources (supporting), writing – review and editing (supporting). **Petra Quillfeldt:** conceptualization (lead), data curation (equal), funding acquisition (equal), investigation (equal), project administration (equal), resources (equal), supervision (lead), writing – review and editing (supporting). **Jochen Bellebaum:** conceptualization (lead), funding acquisition (lead), project administration (supporting), supervision (supporting), validation (lead), writing – review and editing (equal).

## Ethics Statement

All animal work was performed in conformity with Article 44 of the Federal Law of the Russian Federation of 04.24.1995 NO 52‐FZ ‘On Animal Wildlife’.

## Conflicts of Interest

The authors declare no conflicts of interest.

## Supporting information


**Appendix S1.** Supporting Information.

## Data Availability

All data required to reproduce the results of this study are uploaded as part of the supporting S1 (https://doi.org/10.57782/M8NAJ2) ‘dataset folder.zip’ (temporary download‐link until publication: https://cloudsync.uol.de/s/8HsZXJb26tByCP9) including the following files: ‘migration.characteristics.LTD.xlsx’: table containing breeding status, staging timings, longitude and latitudes for staging periods. ‘LTD_GeoLight.xlsx’: locations obtained from the GeoLight Model. ‘LTD_probGLS.xlsx’: locations obtained from the probGLS Model. ‘GLS raw data’—folder containing everything needed to restart an analysis of the light‐level geolocator data: ‘GLS deploy + ringing table.xlsx’ + folder with lux files of every geolocator containing the light data. For geolocators whose internal clock was still running at the time of retrieval, an additional lux file with the ending ‘_driftadj’ is available, correcting for the internal time drift of the geolocator, resulting in more accurate timings. A folder containing deg‐files, in which water conductivity, wet count and temperature values are stored.
