# Supplementary material for: Individual Variation in Migration and Wintering Patterns of Long‐Tailed Ducks Clangula hyemalis From a Population in Decline
Source: Ecol Evol. 2025 Apr 1;15(4):e71187. doi: 10.1002/ece3.71187 (PMC11961394; doi:10.1002/ece3.71187)

**Appendix** to Karwinkel, Pollet et al. 2025 “Individual variation in migration and wintering patterns of long-tailed ducks *Clangula hyemalis* from a population in decline” Ecology and Evolution

DOI: 10.1002/ece3.71187

**FIGURE S1 |** Example plots of one individual used for the manually defining of the pre-breeding stage: A) relative conductivity (blue circles) and relative wet counts (red crosses) and B) conductivity (blue circles, same as in A) and temperature (red line) values over time. black line corresponds to a conductivity of 115, separating brackish water from the Baltic Sea (< 115) and salt water (> 115); Horizontal red line corresponds to a wet count of 360 (18 h per day on the water). Time starts in may during the wintering sage in the Baltic indicated by conductivity slightly <115 (below black line), onset of spring migration is indicated by a drop in conductivity and drop of wet count (probably due to flying time). Pre-breeding stage start is indicated by conductivity values >115 (above black line) indicating saltwater and the drop of temperature, most likely in the Arctic ocean. The start of the breeding stage is indicated by the dramatic drop of conductivity values to <40, indicating inland freshwater lakes. Active breeding (end of June/beginning of July) is indicated by a drop in wet counts and a constant rise in temperature.

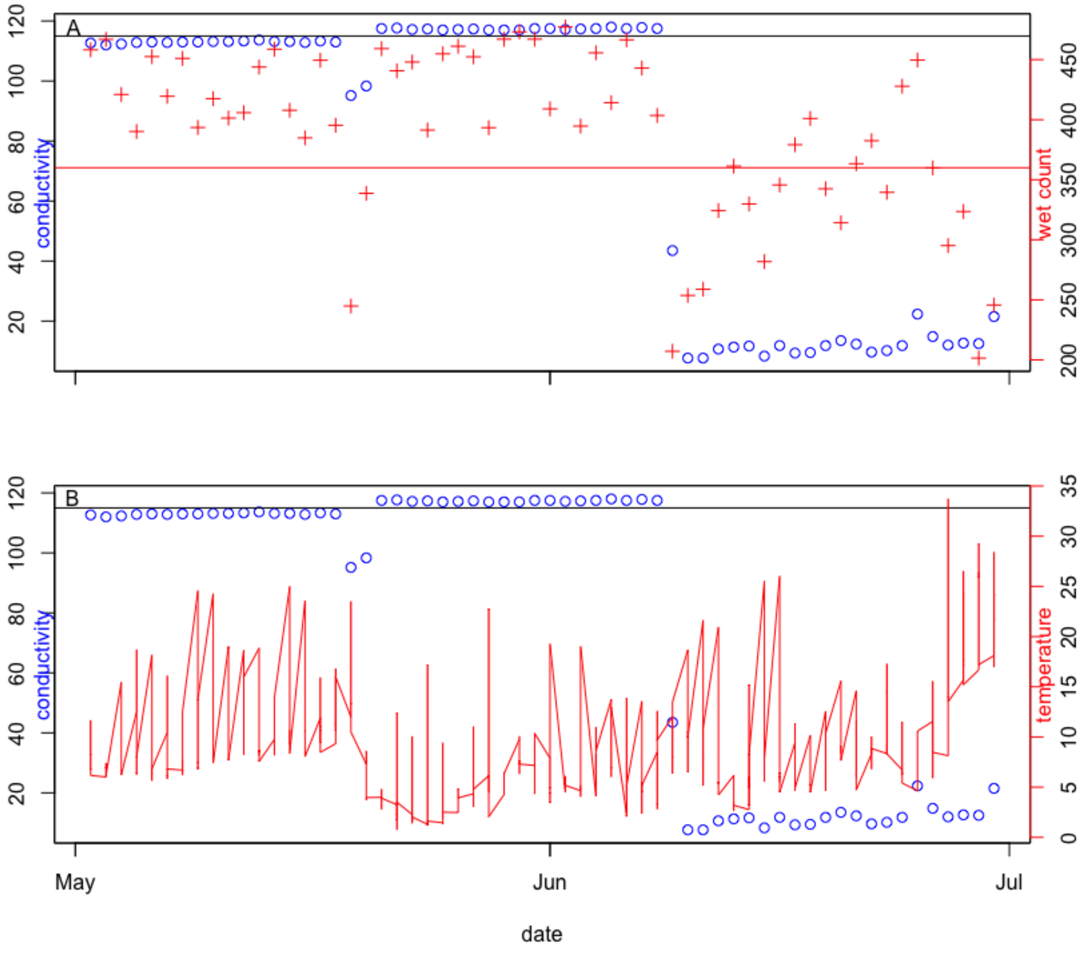

**FIGURE S2 |** Average winter water conductivity for 2017 (red) and 2018 (green) and 2019 (blue) for the tracked female long-tailed ducks including the month December to February. The vertical line at value 115 in separates brackish water (< 115) from salt water (> 115). Conductivity values are relative values between 0 and 128 as derived from the geolocators.

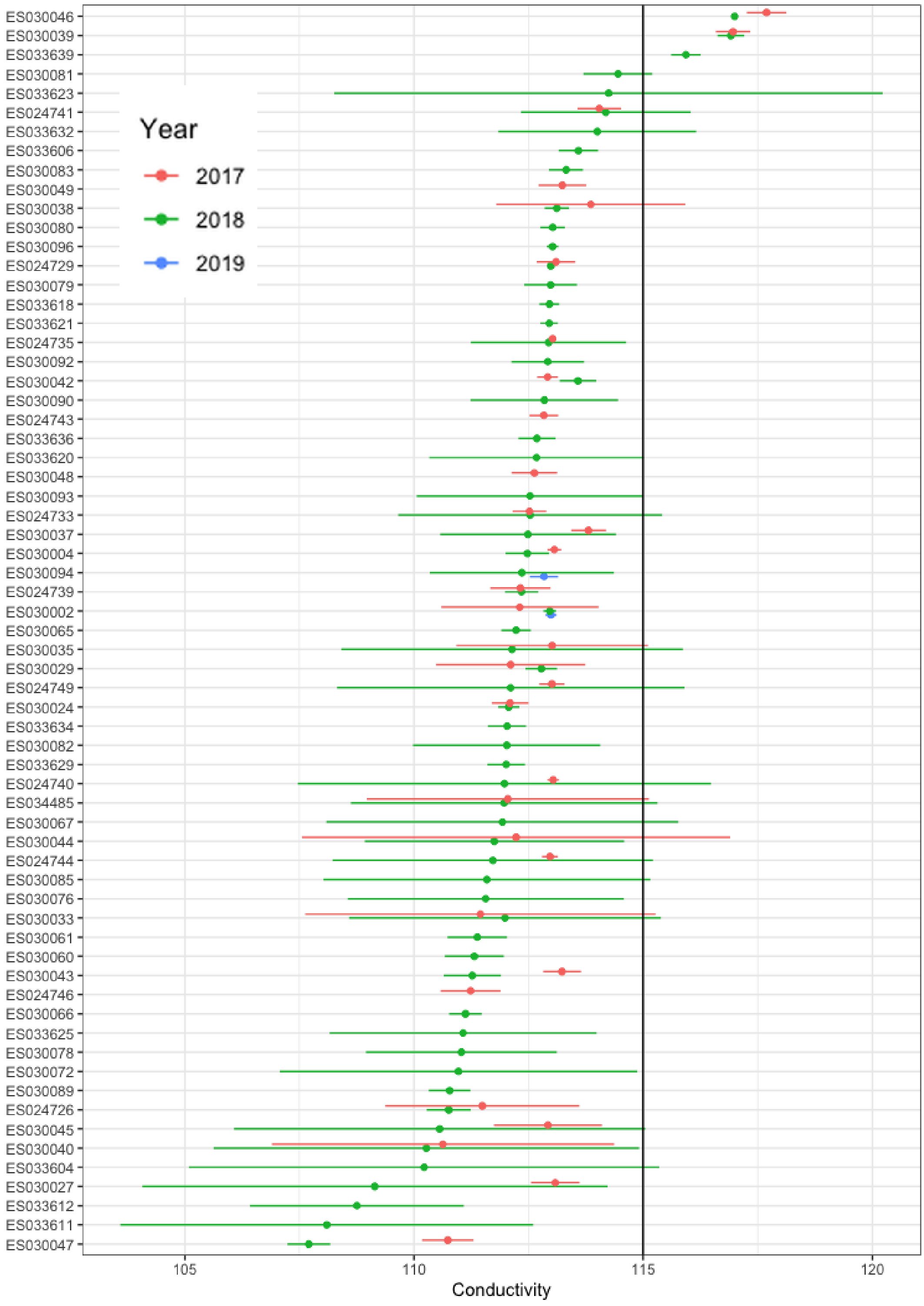

**TABLE S1 |** Summary of repeatability models of different traits from year-round female long-tailed duck tracking. The factor breeding status (see method) was included in the first three models, whereas the rest of the models were calculated with year as fixed effect. Sample size (n) is given as number of year-round tracks with the number of individuals in brackets as several individuals were tracked over two years.

| trait | factors included in model | R ± standard error | 95 % confidence interval | p-value | n Number of tracks (individuals) |
|---|---|---|---|---|---|
| departure date from breeding area | breeding status | 0.833±0.055 | 0.699-0.915 | 0.001 | 93(64) |
| duration post-breeding stage | breeding status | 0.502±0.139 | 0.2-0.736 | 0.004 | 93(64) |
| longitude post-breeding stage | breeding status | 0.827±0.061 | 0.691-0.916 | 0.003 | 90(64) |
| arrival date at wintering stage | year | 0.444±0.148 | 0.117-0.683 | 0.024 | 93(64) |
| longitude November | year | 0.927±0.023 | 0.875-0.963 | 0.001 | 94(65) |
| longitude December | year | 0.967±0.011 | 0.941-0.983 | 0.001 | 94(65) |
| longitude January | year | 0.973±0.009 | 0.952-0.986 | 0.001 | 94(65) |
| longitude February | year | 0.972±0.009 | 0.951-0.986 | 0.001 | 94(65) |
| longitude March | year | 0.945±0.019 | 0.901-0.972 | 0.001 | 93(64) |
| longitude April | year | 0.916±0.027 | 0.854-0.958 | 0.001 | 90(62) |
| departure date from wintering stage | year | 0.773±0.073 | 0.604-0.888 | 0.001 | 89(62) |
| duration of spring migration | year | 0.469±0.16 | 0.097-0.725 | 0.016 | 83(59) |
| arrival at breeding stage | year | 0.42±0.157 | 0.063-0.68 | 0.030 | 89(62) |

**FIGURE S3 |** Intra-individual repeatability of migratory traits, derived from a year-round geolocator tracking of female long-tailed ducks. Dots represent repeatability values, lines their standard error. Colours indicate different co-factors in the repeatability model: black: no co-factors; red: year as co-factor; blue: breeding success as co-factor. The numbers behind the trait names represents the number of year-round tracks, the number in brackets represents the number of different individuals from which those tracks were derived.

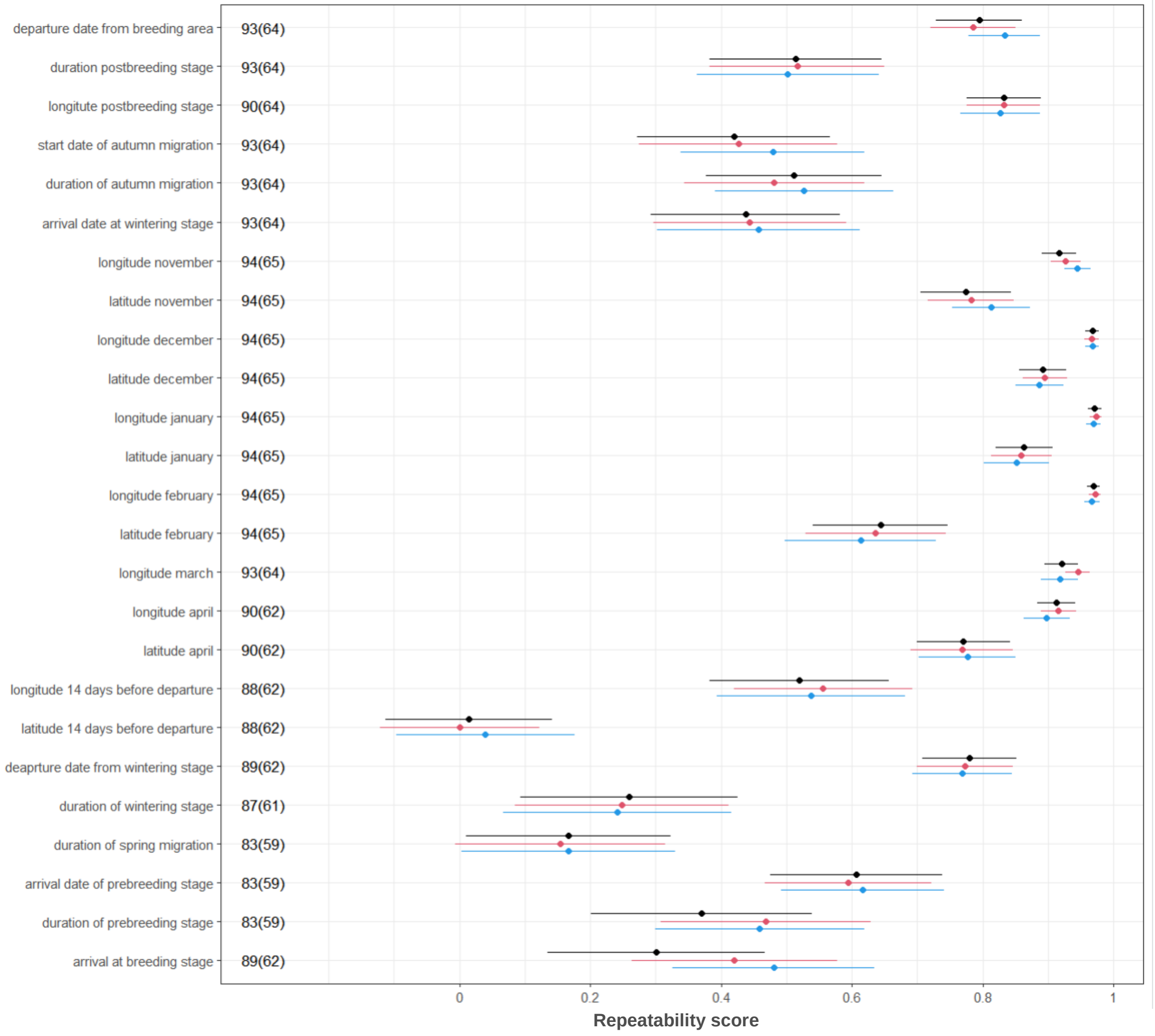

**FIGURE S4 |** Longitudes (mean ± SD) of post-breeding staging areas of migrating female long-tailed ducks from the probGLS model. Plus symbols (+) indicate breeding area longitudes.

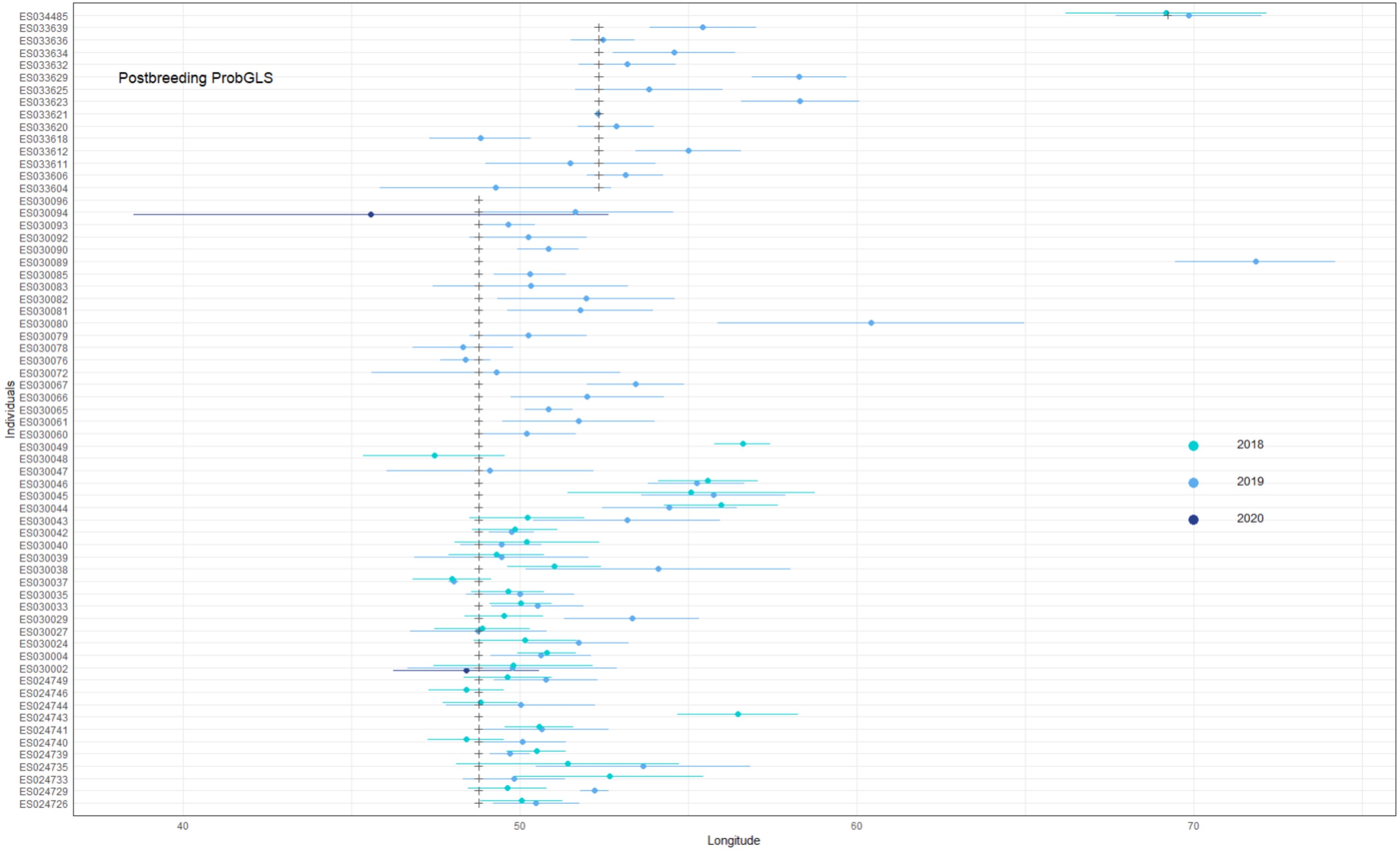

**FIGURE S5 |** Longitudes (mean ± SD) of pre-breeding staging areas of migrating female long-tailed ducks from the probGLS model. Plus symbols (+) indicate breeding area longitudes.

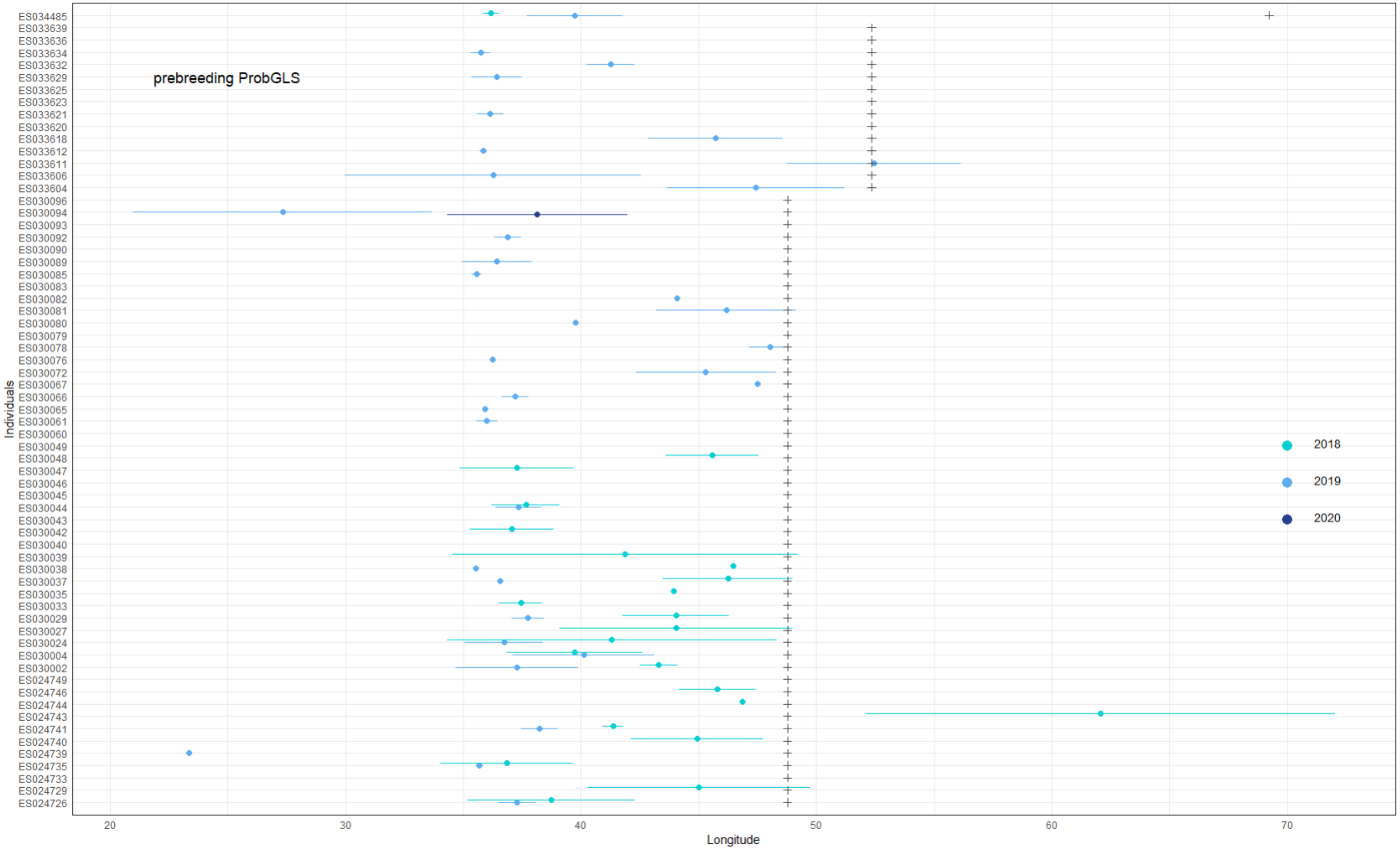

**FIGURE S6 |** Longitudes (mean ± SD) of wintering stage of migrating female long-tailed ducks from the GeoLight model. Plus symbols (+) indicate breeding area longitudes.

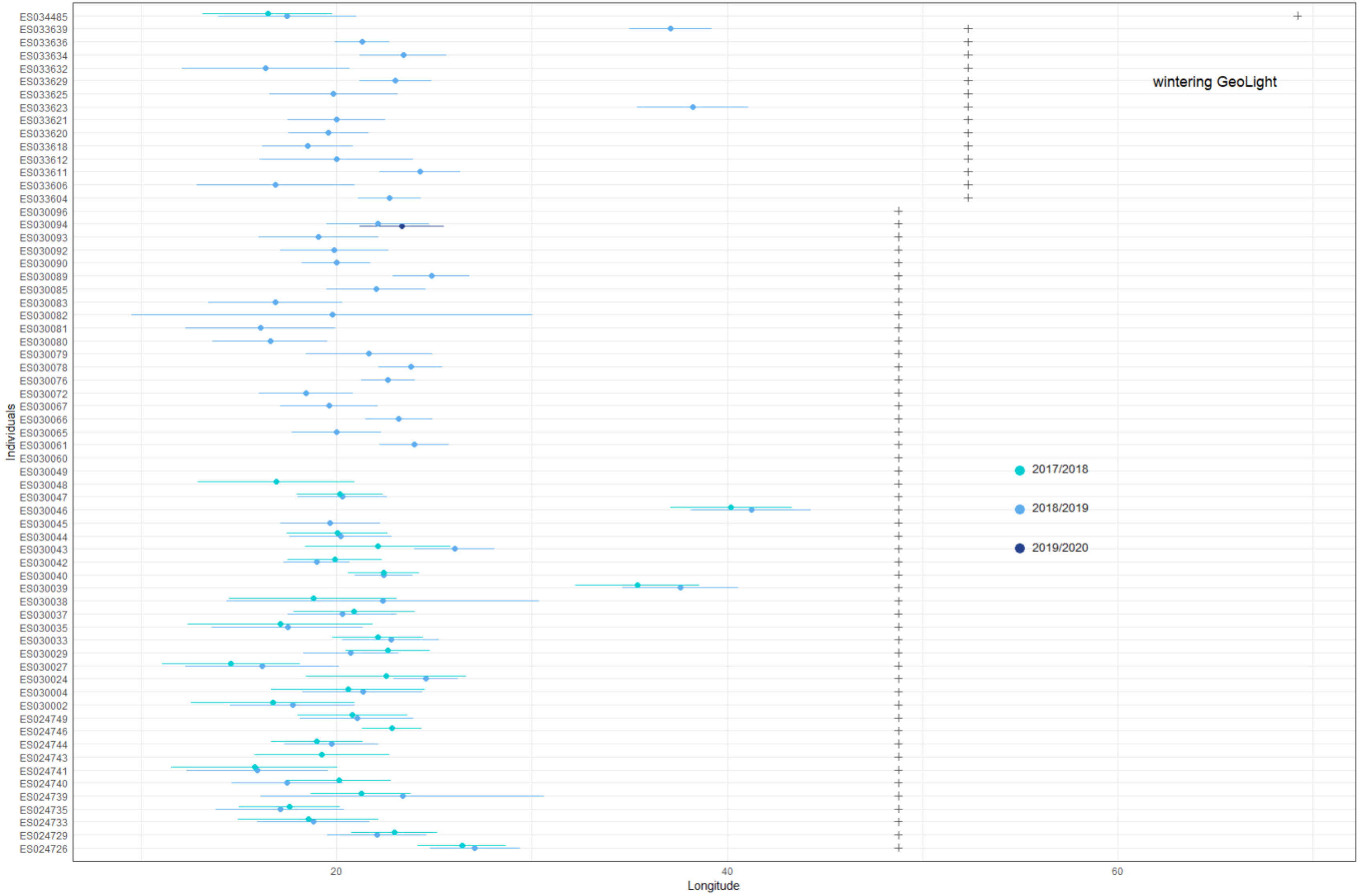

**FIGURE S7 |** Longitudes (mean ± SD) of wintering stage of migrating female long-tailed ducks from the probGLS model. Plus symbols (+) indicate breeding area longitudes.

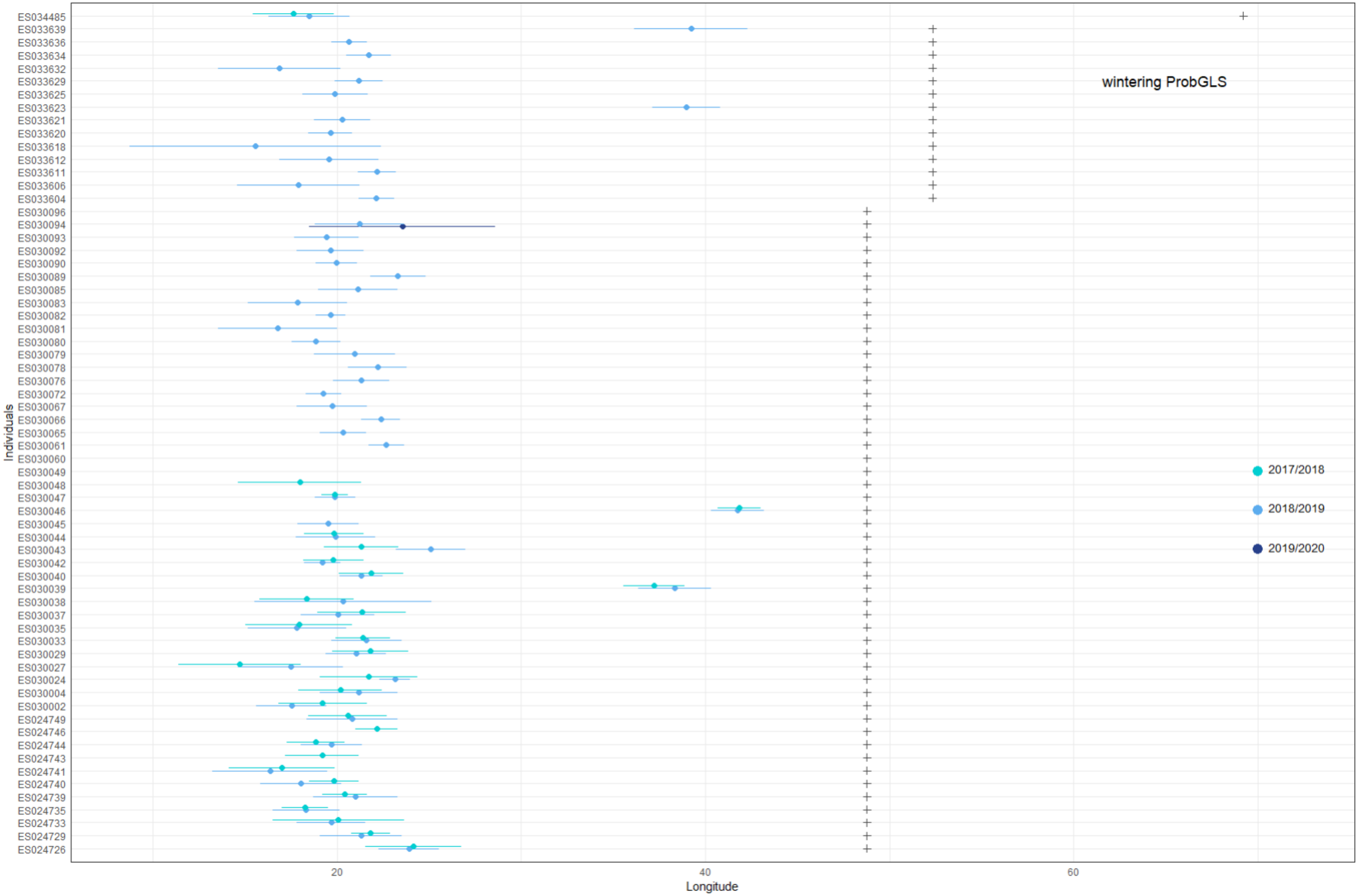

Supplement: Supplementary file 1 — Appendix S1. Supporting Information. [file ECE3-15-e71187-s001.pdf]
